# Effect of a Concise Educational Program on COVID-19 Vaccination Attitudes

**DOI:** 10.3389/fpubh.2021.767447

**Published:** 2021-11-30

**Authors:** Arielle Kaim, Maya Siman-Tov, Eli Jaffe, Bruria Adini

**Affiliations:** ^1^Department of Emergency and Disaster Management, Faculty of Medicine, School of Public Health, Sackler Tel Aviv University, Tel Aviv, Israel; ^2^Israel National Center for Trauma and Emergency Medicine Research, Sheba Medical Center, The Gertner Institute for Epidemiology and Health Policy Research, Ramat-Gan, Israel; ^3^Public Relations, Training and Volunteers Division, Magen David Adom, Tel Aviv, Israel

**Keywords:** uptake, compliance, vaccination attitudes, educational intervention, pandemic

## Abstract

**Background:** Vaccination has been recognized as a vital step for containing the COVID-19 outbreak. To ensure the success of immunization efforts as a public health containment measure, a high level of public vaccination compliance is essential. Targeted educational programs can be utilized to improve attitudes toward vaccination and improve the public's uptake of protective measures.

**Methods:** In this cross-sectional study, we aimed to evaluate the impact of a concise educational program on perceived knowledge regarding the COVID-19 vaccine, vaccine importance and trust, protection and fear from COVID-19, trust in authorities, as well as individual resilience.

**Results:** The study evaluated 503 participants that completed the questionnaire before and after viewing a concise video tutorial on vaccination. Following the educational program, scores of five variables increased significantly compared to their pre-viewing level: knowledge, personal resilience, trust in authorities, vaccine importance, as well as perceived protection. Those that were vaccinated and/or intend to be vaccinated (*N* = 394) report higher levels of knowledge, trust in authorities, vaccine importance, vaccine trust, and fear of being infected as compared to those that are unwilling to get vaccinated. Positive significant correlations were found between resilience and trust in authorities (*r* = 0.169, *p* < 0.001), vaccine importance (*r* = 0.098, *p* = 0.028), and feeling protected (*r* = 0.310, *p* < 0.001). Trust in authorities was positively correlated with vaccine importance (*r* = 0.589, *p* < 0.001) and vaccine trust (*r* = 0.177, *p* < 0.001). Vaccine importance was positively correlated with vaccine trust (*r* = 0.149, *p* = 0.001), but not correlated with knowledge score.

**Conclusion:** The findings of the study demonstrate the benefits of educational programs on improving attitudes toward vaccination acceptability. Incorporation of such concise educational programs by authorities may improve uptake of COVID-19 vaccination and help overcome public vaccine hesitancy. We recommend that such a concise and easily implementable educational program be incorporated as a response component to the current and future outbreaks.

## Introduction

From its onset in December 2019, the COVID-19 outbreak has resulted in widespread disturbances in health, economic, societal, and political systems worldwide, and has posed unprecedented challenges including massive morbidity and mortality rates (1, 2). As of August 1, 2021, the pandemic has impacted 192 countries and regions, with over 198 million laboratory confirmed cases and over 4.2 million global deaths (3). Given the urgency of the outbreak, the international community quickly mobilized ways to accelerate the research and development of a viable vaccine (4). The World Health Organization emphasized that vaccination would be the most effective way to contain the outbreak and evolve from a state of crisis to a state of normalcy (5). To ensure the success of immunization efforts as a public health containment measure, a high level of public vaccination compliance and acceptance is essential. The goal is to reach a state described as the “herd immunity” phenomenon, which would not only provide direct immunity to those who were vaccinated but also extend toward curtailing infection among individuals who have not received a vaccination (6). Previous investigations on the subject indicate that vaccine availability does not often translate to the uptake of vaccination, but rather researchers have identified inconsistency and variability with regard to vaccination cooperation among general population members (7, 8).

As reported during the 2009 H1N1 pandemic, the acceptance of a novel vaccine showed unsatisfying results, as public willingness of inoculation ranged from 17 to 67% across Europe, America and Australia (9–11). Of note, while this vaccine was developed *ad-hoc* for pandemic containment purposes, Influenza is not considered a novel disease by the public. Nevertheless, convincing the public to accept the vaccine proved difficult and many vials purchased were discarded without being used during the H1N1 pandemic.

Vaccine skepticism or vaccine hesitancy continues to be a major hurdle for overall population inoculation during the COVID-19 outbreak (12, 13). Without such considerations, high rates of non-compliance will undermine the possible benefits of herd immunity during the COVID-19 pandemic (6). Lessons from previous infectious disease pandemics, indicate that motivators and obstacles to inoculation compliance are manyfold for a novel vaccine. In the United States, the findings from the H1N1 influenza (or Swine flu) pandemic by Quinn et al. (13) indicate that improved acceptance of a vaccine is impacted by previous history of accepting the seasonal influenza vaccine and reduced concern about the safety of the vaccine (13). Correspondingly, findings by Seale et al. (14) conclude that improved intention to be vaccinated is associated with a higher perceived risk of developing the H1N1 influenza and an increased perceived severity of the pandemic (14). Furthermore, the researchers conclude that individuals were motivated to uptake vaccination, dependent on the personal protection they felt the vaccination would provide them or the perceived efficacy of the vaccine; fear of side effects and safety concerns were associated with a weakened intention to be vaccinated (14). Wariness toward the safety of the vaccine stems to a large extent from the speed of its development (12). Dror et al. (12) indicate that the greatest concern with regard to the COVID-19 vaccine, for both medical personnel and the general public, are fears of the vaccine's safety. These concerns result from the accelerated vaccine development, with the primary safety considerations noted are quality control, potential side effects, and associated COVID-19 illness (12).

Several studies during the H1N1 pandemic among healthcare workers established that social influences were also at play in the acquiescence or apprehension toward vaccination. If it was perceived that others (such as family, friends, and colleagues) want you to be vaccinated or have already been vaccinated themselves, this was positively associated with the intention to be vaccinated (15, 16). It was also presented that the source of information plays an important role in shaping the knowledge and motivating vaccination compliance. Higher levels of knowledge are associated with enhanced vaccination uptake (17). Individuals who obtained information about the vaccination from their healthcare provider or public health department were found to have an improved uptake of vaccination (18). Conversely, a study in Turkey found that those that accessed information from non-official sources (such as emails or mass media sources) were less likely to have been vaccinated (19). Lastly, an additional element that has been identified to impact the decision-making process for uptake of control measures is perceived individual resilience, which has been associated with increased education and perceived knowledge (20).

Thus, it is essential to identify measures that can positively impact on vaccine uptake, so as to enhance the capacity to contain pandemics. In this study, we evaluate the effect of a concise educational program on perceived knowledge regarding the COVID-19 vaccine, perceived vaccine importance and trust, perceived protection and fear from COVID-19, perceived trust in authorities, and lastly, individual resilience. We focus our analysis specifically on the state of Israel. Short educational programs have already been successfully shown to provide an easily implementable and effective way to educate and empower the public concerning understanding of emerging risks (20). The present study strived to identify whether such measures may also contribute to improve uptake of compliance to vital protective measures, such as novel vaccinations. A substantial proportion of the global population is apprehensive concerning such measures and does not comply with the call to be vaccinated. As vaccinations are an important component of containing the pandemic, this study may have an important contribution to the utilization of steps designated to elevate such a compliance.

## Methods

### Study Design

An interventional study was conducted in January 2021, in the midst of the COVID-19 outbreak, at the initial phase of vaccinating the population against the virus. The study was based on subjecting a representative sample of the Israeli population to a short informative video tutorial that focused on assorted elements regarding the COVID-19 vaccination.

The research assessed attitudes toward the vaccine before and immediately after watching a concise (6 min) video-film, by responding to the same questionnaire at both times.

The responses were collected by the iPanel agency, that is the biggest internet panel company in Israel, that comprises of more than 100,000 respondents from the diverse demographic and geographical groups (http://www.ipanel.co.il). Since 2006, this company provides an operational platform for a wide variety of information collection services that maintains the strict standards of the European Society for Opinion and Marketing Research (ESOMAR). In line with the official data issued by the Israeli Central Bureau of Statistics, and to ensure an appropriate representation of the Israeli's population concerning age, gender, religiosity, place of residence and additional demographics, the sample population was defined, utilizing stratified sampling technique. The use of internet panels has greatly expanded in the past decade, after their validity has been shown broadly (21).

### Intervention

Based on interventional mapping (IM), the short educational program was based on a short 6 min video tutorial that was prepared following the development of the COVID-19 vaccine. The target population was defined as Israeli adults, as they were authorized to receive the vaccine. The tutorial aimed to influence and boost their knowledge and understanding of the COVID-19 vaccine. The video was specifically directed to relay vital information to the public concerning the characteristics of the COVID-19 vaccine, its development, its potential side-effects, conspiracy correction about the vaccine, and respective health guidelines and recommendations. The making of the educational tool was done by an expert team, proficient in the development of training programs. The information in the short tutorial was presented by a male paramedic while dressed in the official uniform of the National Israeli Emergency Medical Services (Magen David Adom, MDA). MDA is considered a highly prestigious foundation, that is highly trusted by most sectors of the Israeli public (22). Routinely, MDA is the Israeli pre-hospital organization serving a population of 9 million. During the pandemic, it was heavily employed in the fight against the COVID-19 outbreak- among its work during this time was the distribution of concise educational materials via social media to promote the vaccination campaign process (23).

The framework of the conducted research included a literature review → the design of the short educational video tutorial → distribution of the assessment tool, that was filled for the first time by the sample of the internet panel → watching the short video tutorial → and lastly, the second filling of the assessment tool.

Such a framework was formerly used by the researchers of the present study in another study that examined the impact of a brief educational intervention on the knowledge, perceived knowledge, safety and resilience of the Israeli public during COVID-19 crisis (20). The two studies, though conducted via the same internet panel provider and focusing on the same target population (the Israeli society), included two different, independent samples of respondents.

### Participants

The sample size was computed based on OpenEpi (https://www.openepi.com/SampleSize), requiring 385 participants. A representative sample of 503 respondents participated in all three phases of the study (before and after watching the film), after submitting their informed consent. The study was approved by the Ethics Committee of the Tel Aviv University (# 0002588-1 from January 6, 2021). All the responses were accumulated anonymously, without any identifiable information collected.

### The Study Tool

The questionnaire included a short introduction, which specified background information, the goals of the study, its methodology, the ability to stop participation at any time, and the strict adherence to anonymity and confidentiality.

The questionnaire consisted of 10 parts, based on measures that were developed explicitly for this study, with the exception of individual resilience, which was based on a previously validated tool by Connor and Davidson [Connor-Davidson Resilience Scale (CD-RISC)]. Content validity of the questionnaire was achieved through its examination by six content experts and its pilot testing with participation of 20 individuals, prior to the dissemination stage. The components of the questionnaire consisted of the following: (1) perceived knowledge assessed by three items encompassing characteristics of COVID-19, signs and symptoms after vaccination, proficiency of guidelines, and confidence in one's knowledge. The items were rated by a 5-point Likert scale, scaling from 1 = completely disagree to 5 = completely agree. Cronbach's Alpha for the perceived knowledge index was α = 0.654 and α = 0.692 for pre-post educational program, respectively; (2) perceived personal resilience portraying individual feelings of ability and strength in adaptation and recovery from illness, measured by two items ranked by a 5 point Likert scale, ranging from 1 = completely disagree, to 5 = completely agree (24); (3) perceived coronavirus fear assessed by two items; the items were ranked by a 5 point Likert scale, ranging from 1 = completely disagree, to 5 = completely agree; (4) impact of exposure to information published by sources (national ambulance service, local authority, media, social media, ministry of health, coronavirus ambassadors, family and friends, other) about the corona virus on willingness to be vaccinated; measured by a scale ranging from 1 = did not influence at all, to 5 = very influenced or “I have not been exposed to such information”; (5) perception of importance of vaccine assessed by two items, encompassing reasons attributed to preserving the health of family or loved ones, or because family members intend to get vaccinated, on a 5 point Likert scale, ranging from 1 = completely disagree, to 5 = completely agree; (6) trust in information published by authorities concerning the effectiveness and safety of the corona vaccine measured by one item, on a 5 point Likert scale, ranging from 1 = completely disagree, to 5 = completely agree; (7) perceived individual fear of vaccination measured by one item; a 5 point Likert scale, ranging from 1 = completely disagree, to 5 = completely agree; (8) vaccination status measured by one multiple choice item (Already vaccinated or intend to be vaccinated vs. have no intention to be vaccinated); (9) individual intention to be vaccinated measured by one item on a 5 point Likert scale, ranging from 1 = completely disagree, to 5 = completely agree; (10) demographics, assessed by 10 items including gender, age, place of residence, marital status, number of children, number of dependents, education, religion, degree of religiosity, and level of income.

### Statistical Analysis

The participants' demographic characteristics were presented by descriptive statistics (frequency, mean, and standard deviation). Respondents that were vaccinated or intend to be vaccinated were calculated as one group (hereto referred as vaccinated) vs. those who have no intention to be vaccinated. The differences, percentage of change (T2 – T1)/T1 × 100% and the effect sizes of the differences between T1 (prior to the watching the video-tutorial) and T2 (after watching the video-tutorial) were examined by the Paired samples *t*-test. The relationships between knowledge score, resilience, trust in authorities, and personal resilience for COVID-19 vaccination at T1 were analyzed based on Pearson correlation tests The differences in attitudes between the vaccination statuses (vaccinated or not vaccinated) at T1 were analyzed using the independent sample *t*-test. Statistical analyses were conducted using SPSS software version 25. *P*-values lower than 0.05 were considered to be statistically significant.

## Results

The study evaluated 503 participants (presented in Table 1) that completed the questionnaire before and after viewing a concise video tutorial on vaccination. The study group was 50.1% male, with a mean age of 43.81 (± 15.84 SD), ranging from 19 to 85 years. Approximately 45% of the participants characterized themselves as secular, while 22% reported being religious or ultra-religious. These classifications are similar to the overall Israeli population. Most (72%) have over 12 years of education (vocational or academic degree), 45% earn less than the average income while 22% earn above it, and half (56%) of the participants identified themselves as being in a relationship and having children.

**Table 1:** Characteristics of study population (*N* = 503).

	***N* (%)**
**Age**	
Mean ± standard deviation	43.81 ± 15.84
Min-max	(19–85)
**Gender**	
Male	252 (50.1%)
Female	251 (49.9%)
**Religiosity**	
Secular	230 (45.7%)
Traditional	161 (32.0%)
Religious	110 (22.3%)
**Family status**	
In relation without children	78 (15.5%)
In relation with children	281 (55.9%)
No relation no children	114 (22.7%)
No relation with children	30 (6.0%)
**Number of children bellow 18**	
None	199 (39.6%)
1–3	275 (54.7%)
4+	25 (5.7%)
**Level of income**	
Much below mean	141 (28.0%)
Below mean	84 (16.7%)
Mean	132 (26.2%)
Above mean	87 (17.3%)
Much above mean	21 (4.2%)
Refuse to answer	38 (7.6%)
**Level of education**	
≤8 years	7 (1.4%)
9–12 years	136 (27.0%)
Professional	141 (28.0%)
Academic	219 (43.5%)

As presented in Table 2, following the educational program, scores of five variables that were examined in the study increased significantly after viewing the COVID-19 vaccination video, compared to their previous (pre-viewing) level: knowledge (*p* < 0.001), personal resilience (*p* < 0.001), trust in authorities (*p* = 0.002), vaccine importance (*p* = 0.002), as well as perceived protection (*p* < 0.001). The most substantial change was in perceived protection, presenting an effect size of 0.15, and an increase of 6.2% (from 2.75 to 2.92). Knowledge and resilience also increased by 2.4 and 2.5% respectfully, with both an effect size of 0.11.

**Table 2:** Average scores before and after viewing the vaccination video.

	**Before**	**After**	**Δ**	Effect size^*^ (Cohen's d)^**^	***t* (df)**	***p*-value**
Knowledge	3.73 ± 0.75	3.81 ± 0.70	+2.4%	0.11	−6.36 (502)	<0.001
Resilience	3.59 ± 0.79	3.68 ± 0.78	+2.5%	0.11	−3.86 (502)	<0.001
Trust in authorities	3.71 ±0.85	3.76 ± 0.87	+1.3%	0.06	−3.15 (502)	0.002
Vaccine importance	3.69 ± 1.08	3.75 ± 1.00	+1.6%	0.06	−3.17 (502)	0.002
Vaccine trust	3.07 ± 0.85	3.05 ± 0.85	−0.6%	-	1.36 (502)	0.173
Feeling protected	2.75 ± 1.13	2.92 ± 1.08	+6.2%	0.15	−4.15 (502)	<0.001
I'm afraid to be infected	3.57 ± 1.19	3.50 ± 1.13	+2.0%	-	1.85 (502)	0.065

*Data are presented as mean ± standard deviation and median (Q25–Q75). p-value is based on paired sample t-test*.

Effect size *: *0.2-Small, 0.5-Medium, and 0.8-Large*.

Cohen's d **: *appropriate effect size for the comparison between two means*.

Differences in attitudes between the varied vaccination statuses at T1 are presented in Table 3. Those that were vaccinated and/or intend to be vaccinated (*N* = 394) report higher levels of knowledge, trust in authorities, vaccine importance, vaccine trust, and fear of being infected as compared to those that are unwilling to get vaccinated. The most substantial attitude difference between those that were or are scheduled to be vaccinated and those that are not, is concerning the vaccine importance with an effect size of 1.41. Knowledge and trust in authorities also indicated substantial disparities in attitudes between the groups, with effect sizes of 1.07 and 1.04 respectfully.

**Table 3:** Differences in attitudes between those vaccinated/scheduled to be vaccinated vs. not vaccinated (T1).

	**Vaccinated *N* = 394**	**No intent of vaccination *N* = 109**	**Δ**	Effect size^*^ (Cohen's d)^**^	***t* (df)**	***p*-value**
Knowledge	3.89 ± 0.67	3.14 ± 0.73	+23.5%	1.07	−10.2 (501)	<0.001
Resilience	3.60 ± 0.78	3.53 ± 0.82	+2.0%	-	−0.89 (501)	0.373
Trust in authorities	3.90± 0.71	3.02 ± 0.96	+29.0%	1.04	−8.86 (501)	<0.001
Vaccine importance	2.14 ± 0.74	1.12 ± 0.70	+91.1%	1.41	−25.89 (501)	<0.001
Vaccine trust	3.13 ± 0.85	2.85 ± 0.84	+9.8%	0.33	−3.07 (501)	0.002
Feeling protected	2.72 ± 1.12	2.86 ± 1.14	−4.9%	0.12	1.19 (501)	0.232
Afraid to be infected	3.63 ± 1.16	3.33 ± 1.28	+9.0%	0.25	−2.38 (501)	0.018

*Data are presented as mean ± standard deviation and median (Q25–Q75). p-value is based on an independent sample t-test*.

Effect size *: *0.2-Small, 0.5-Medium, and 0.8-Large*.

Cohen's d **: *appropriate effect size for the comparison between two means*.

Before the educational program, a significant positive correlation was found between perceived knowledge and personal resilience (*r* = 0.241, *p* < 0.001), trust in authorities (*r* = 0.468, *p* < 0.001), vaccine importance (*r* = 0.606, *p* < 0.001), vaccine trust (*r* = 0.106, *p* < 0.017) and feeling protected (*r* = 0.189, *p* < 0.001). Positive significant correlations were found between resilience and trust in authorities (*r* = 0.169, *p* < 0.001), vaccine importance (*r* = 0.098, *p* = 0.028), and feeling protected (*r* = 0.310, *p* < 0.001). Trust in authorities was positively correlated with vaccine importance (*r* = 0.589, *p* < 0.001) and vaccine trust (*r* = 0.177, *p* < 0.001). Lastly, vaccine importance was positively correlated with vaccine trust (*r* = 0.149, *p* = 0.001), but not correlated with knowledge score. The correlations are presented in Table 4.

**Table 4:** Pearson correlation between attitudes at T1.

	**Resilience**	**Trust in authorities**	**Vaccine importance**	**Vaccine trust**	**Feeling protected**
Perceived knowledge	*r* = 0.241 *p* < 0.001	*r* = 0.468 *p* < 0.001	*r* = 0.606 *p* < 0.001	*r* = 0.106 *p* = 0.017	*r* = 0.189 *p* < 0.001
Resilience	1	*r* = 0.169 *p* < 0.001	*r* = 0.098 *p* = 0.028	*r* = −0.127 *p* = 0.004	*t* = 0.310 *p* < 0.001
Trust in authorities		1	*r* = 0.589 *p* < 0.001	*r* = 0.177 *p* < 0.001	*r* = 0.050 *p* = 0.259
Vaccine importance			1	*r* = 0.149 *p* = 0.001	*r* = 0.065 *p* = 0.144
Vaccine trust				1	*r* = −0.217 *p* < 0.001
Feeling protected					1
I'm afraid from infected	*r* = −0.175 *p* < 0.001	*r* = 0.153 *p* = 0.001	*r* = 0.078 *p* = 0.079	*r* = 0.396 *p* < 0.001	*r* = −358 *p* < 0.001

The results indicate a positive significant correlation between age and knowledge (*r* = 0.334, *p* < 0.001), trust in authorities (*r* = 0.189, *p* < 0.001) and vaccine importance (*r* = 0.343, *p* < 0.001). Regarding gender, males compared to females reported a higher level of knowledge (3.85 vs. 3.61 *p* = 0.001), resilience (3.73 vs. 3.44 *p* < 0.001), vaccine importance (3.88 vs. 3.50 *p* < 0.001) and feeling protected (2.95 vs. 2.55 *p* < 0.001). Accordingly, they reported lower levels of feeling concerned (3.38 vs. 3.75 *p* < 0.001). Participants with higher levels of income reported higher levels of resilience compared to those with medium and lower levels (3.77 vs. 3.59 and 3.52, respectively *p* = 0.035). The same trend was seen for vaccine importance (4.04 vs. 3.65 and 3.60 *p* = 0.001). Significant differences were observed among lower and higher levels of income, both for level of resilience and for vaccine importance.

## Discussion

Widespread uptake of COVID-19 vaccines worldwide is a crucial step for moving forward from the state of crisis resulting from the current COVID-19 pandemic (5). As the success of immunization programs relies highly on extensive public vaccination acceptance, it is critical to develop and identify educational programs which may impact productively on vaccine uptake, in order to enhance the capacity to contain pandemics such as the current outbreak (6). Herein, we evaluate the impact of a concise educational problem on perceived knowledge regarding the COVID-19 vaccine, perceived vaccine importance and trust, perceived protection and fear from COVID-19, individual resilience, and lastly, perceived trust in authorities.

The findings of this study demonstrate a significant overall increase in five (perceived knowledge, individual resilience, perceived trust in authorities, perceived vaccine importance, and feeling protected) examined variables immediately following the educational program, to varying degrees of effect sizes. The findings of this study are in line with previous research involving educational programs in which short videos have been shown to be an effective method for improving knowledge, attitudes, and health behavior (20, 25–27). Goodman et al. found that video education positively improved health beliefs about vaccination against influenza (26), while Drokow et al. (27) indicated that video education is influential in improving actual uptake of HPV vaccines (27).

The study also identified that there are substantial differences in perceived levels of knowledge, trust in the authorities, vaccine importance and trust, and fear of being infected amongst those who are vaccinated (or intend to be vaccinated) and those that do not intend to get vaccinated. These differences are in line with previous findings which determined that higher levels of knowledge (28, 29), trust (30–32), and fear of infection (29, 33, 34) are important determinants for vaccine uptake.

The study identified several positive associations between examined variables which coincide with the findings above. Particularly important are the findings which present the significant relationships between trust in authorities, perceived knowledge about the vaccine and personal resilience with deemed vaccine importance. The positive associations that were found in the study are of utmost importance, as they provide targetable factors which may impact on the uptake of protective behavior. In order to shape compliance to preventive behavior such as vaccination, authorities ought to consider making use of health communication strategies within the context of short interventional tools (such as the one utilized in this study) which aim to improve knowledge of the public regarding the vaccine, personal resilience, trust as well as convey that the war against COVID-19 is not yet over which may affect the perceived fear of the public toward infection. The outcomes of this research exhibit a cost-effective and quickly implementable research intervention design, which allows for the proactive and speedy dissemination of directed and tailorable material to meet particular objectives during emergencies and crisis.

Additional results of this study expose several demographic factors that are associated with the evaluated variables. For example, males and those with higher levels of income reported higher levels of vaccine importance. These findings support conclusions from previous investigations regarding gender and socio-economic differences in the determinants of willingness to get the COVID-19 vaccine (35–38). While women tend to be more proactive in preventive behaviors, in the context of uptake of vaccination they are often less willing than men and this may be attributed to evidence that women are more cautious about risks and take longer to make decisions (39). Given the novelty of the vaccine, this may be a contributing factor to such caution (12). Similarly, as expected regarding socio-economic status, immunization rates are often higher among those who are wealthier and more educated (40). These findings provide valuable insights into identifying various target groups in need of educational programs regarding COVID-19 vaccination, such as among women. In order to improve the uptake of vaccinations, the facilitation of targeted supplementary education to this demographic group may contribute to improved levels of compliance.

Given that vaccine hesitancy in the context of the COVID-19 pandemic remains a hurdle (12, 13), the findings of the study provide valuable concise training programs which can be utilized to promote behavioral change among public health officials.

Limitations of this work are that it assesses attitudes toward vaccination cross-sectionally when the vaccination rollout campaigns began, even though it is possible that sentiments may adjust as more individuals are inoculated and additional studies are conducted which evaluate the efficacy and safety of such vaccines. In the future, this can be attended to by conducting the study longitudinally. Nonetheless, as understanding the sentiment of the population toward vaccination is vital during the initial stages of the vaccine campaign, there is great value in understanding the public's attitudes. Another limitation is that this study was conducted via the internet in order to ensure quick response collection. While this response methodology allowed for a quick collection of data and ensured a representative sample of the Israeli adult population, the conclusions must be limited to individuals who have high computing skills and access to an internet source. A further limitation is that the assessment of change was conducted directly after the respondents watched the video tutorial; it is vital that the continued effect over time is assessed in future studies. As is the case in all research based on questionnaires, social desirability bias cannot be fully dismissed, including concerning declaration of levels of trust. Nonetheless, as the study was conducted through an internet panel, iPanel that is entirely independent and unconnected to any response entity, the discussed above potential bias is most probably better controlled. Lastly, as this study was conducted in the Hebrew language, members of the Israeli society who are not fluent in the language were excluded from participating. Nevertheless, our findings have valuable implications as we show that an inexpensive and convenient short training programs can be an effective means for improving attitudes toward vaccination and utilized to overcome the vaccine hesitancy hurdles in the COVID-19 context and future such outbreaks. Conducted future research should assess the long-term impacts of such educational programs (such as the one conducted) on actual uptake of protective behavioral practices among study participants.

## Data Availability Statement

The raw data supporting the conclusions of this article will be made available by the authors, without undue reservation.

## Ethics Statement

The studies involving human participants were reviewed and approved by Ethics Committee, Tel Aviv University. The patients/participants provided their written informed consent to participate in this study.

## Author Contributions

EJ: conceptualization, investigation, and resources. EJ and BA: methodology. BA and MS-T: validation. MS-T: formal analysis. AK and MS-T: data curation. AK: writing—original draft preparation. EJ, MS-T, and BA: writing—review and editing. BA: supervision. All authors have read and agreed to the published version of the manuscript.

## Conflict of Interest

The authors declare that the research was conducted in the absence of any commercial or financial relationships that could be construed as a potential conflict of interest.

## Publisher's Note

All claims expressed in this article are solely those of the authors and do not necessarily represent those of their affiliated organizations, or those of the publisher, the editors and the reviewers. Any product that may be evaluated in this article, or claim that may be made by its manufacturer, is not guaranteed or endorsed by the publisher.

## References

[1] Mofijur M Fattah IR Alam MA Islam AS Ong HC Rahman SA. Impact of COVID-19 on the social, economic, environmental and energy domains: lessons learnt from a global pandemic. Sustain Prod Consum. (2021) 26:343–59. 10.1016/j.spc.2020.10.016 33072833 PMC7556229

[2] Shadmi E Chen Y Dourado I Faran-Perach I Furler J Hangoma P. Health equity and COVID-19: global perspectives. Int J Equity Health. (2020) 19:104. 10.1186/s12939-020-01218-z 32586388 PMC7316580

[3] Center for Systems Science and Engineering. COVID-19 Map. John Hopkins Coronavirus Resourse Center (2019).

[4] World Health Organization. COVID 19 Public Health Emergency of International Concern (PHEIC). Global Research and Innovation Forum: Towards a Research Roadmap. Washington, DC: Pan American Health Organization.

[5] Chen W. Promise and challenges in the development of COVID-19 vaccines. Hum Vaccin Immunother. (2020) 16:2604–8. 10.1080/21645515.2020.1787067 32703069 PMC7733917

[6] Dermody TS DiMaio D Enquist LW. Vaccine safety, efficacy, and trust take time. Annu Rev Virol. (2021) 8:iii–iv. 10.1146/annurev-vi-08-102220-100001 33186082

[7] MacDonald NE. Vaccine hesitancy: definition, scope and determinants. Vaccine. (2015) 33:4161–4. 10.1016/j.vaccine.2015.04.036 25896383

[8] Jacobson RM Sauver JL Rutten LJ. Vaccine hesitancy. Mayo Clin Proc. (2015) 90:1562–8. 10.1016/j.mayocp.2015.09.006 26541249

[9] Eastwood K Durrheim DN Jones A Butler M. Acceptance of pandemic (H1N1) 2009 influenza vaccination by the Australian public. Med J Aust. (2010) 192:33–6. 10.5694/j.1326-5377.2010.tb03399.x 20047546

[10] Schwarzinger M Flicoteaux R Cortarenoda S Obadia Y Moatti JP. Low acceptability of A/H1N1 pandemic vaccination in French adult population: did public health policy fuel public dissonance? PLoS ONE. (2010) 5:e10199. 10.1371/journal.pone.0010199 20421908 PMC2856629

[11] Sypsa V Livanios T Psichogiou M Malliori M Tsiodras S Nikolakopoulos I. Public perceptions in relation to intention to receive pandemic influenza vaccination in a random population sample: evidence from a cross-sectional telephone survey. Eurosurveillance. (2009) 14:19437. 10.2807/ese.14.49.19437-en 20003909

[12] Dror AA Eisenbach N Taiber S Morozov NG Mizrachi M Zigron A. Vaccine hesitancy: the next challenge in the fight against COVID-19. Eur J Epidemiol. (2020) 35:775–9. 10.1007/s10654-020-00671-y 32785815 PMC8851308

[13] Quinn SC Kumar S Freimuth VS Kidwell K Musa D. Public willingness to take a vaccine or drug under emergency use authorization during the (2009). H1N1 pandemic. Biosecur Bioterror. (2009) 7:275–90. 10.1089/bsp.2009.0041 19775200 PMC2998968

[14] Seale H Heywood AE McLaws ML Ward KF Lowbridge CP Van D. Why do I need it? I am not at risk! Public perceptions towards the pandemic (H1N1) 2009 vaccine. BMC Infect Dis. (2010) 10:99. 10.1186/1471-2334-10-99 20403201 PMC2864274

[15] Zijtregtop EA Wilschut J Koelma N Van Delden JJ Stolk RP Van Steenbergen J. Which factors are important in adults' uptake of a (pre) pandemic influenza vaccine? Vaccine. (2009) 28:207–27. 10.1016/j.vaccine.2009.09.099 19800997

[16] Lau JT Yeung NC Choi KC Cheng MY Tsui HY Griffiths S. Factors in association with acceptability of A/H1N1 vaccination during the influenza A/H1N1 pandemic phase in the Hong Kong general population. Vaccine. (2010) 28:4632–7. 10.1016/j.vaccine.2010.04.076 20457289 PMC7131323

[17] Tabacchi G Costantino C Cracchiolo M Ferro A Marchese V Napoli G. Information sources and knowledge on vaccination in a population from southern Italy: the ESCULAPIO project. Hum Vaccin Immunother. (2017) 13:339–45. 10.1080/21645515.2017.1264733 28032814 PMC5328217

[18] Hidiroglu S Ay P Topuzoglu A Kalafat C Karavus M. Resistance to vaccination: the attitudes and practices of primary healthcare workers confronting the H1N1 pandemic. Vaccine. (2010) 28:8120–4. 10.1016/j.vaccine.2010.09.104 20950726

[19] Maltezou HC Dedoukou X Patrinos S Maragos A Poufta S Gargalianos P. Determinants of intention to get vaccinated against novel (pandemic) influenza A H1N1 among health-care workers in a nationwide survey. J Infect. (2010) 61:252–8. 10.1016/j.jinf.2010.06.004 20600304

[20] Kaim A Jaffe E Siman-Tov M Khairish E Adini B. Impact of a brief educational intervention on knowledge, perceived knowledge, perceived safety, and resilience of the public during COVID-19 crisis. Int J Environ Res Public Health. (2020) 17:5971. 10.3390/ijerph17165971 32824591 PMC7460211

[21] Hays RD Liu H Kapteyn A. Use of internet panels to conduct surveys. Behav Res Methods. (2015) 47:685–90. 10.3758/s13428-015-0617-9 26170052 PMC4546874

[22] Mizrahi S Vigoda-Gadot E Cohen N. Drivers of trust in emergency organizations networks: the role of readiness, threat perceptions and participation in decision making. Public Manag Rev. (2021) 23:233–53. 10.1080/14719037.2019.1674367

[23] Jaffe E Strugo R Bin E Blustein O Rosenblat I Alpert EA. The role of emergency medical services in containing COVID-19. Am J Emerg Med. (2020) 38:1526–7. 10.1016/j.ajem.2020.04.023 32327247 PMC7165122

[24] Campbell-Sills L Stein MB. Psychometric analysis and refinement of the connor–davidson resilience scale (CD-RISC): validation of a 10-item measure of resilience. J Trauma. (2007) 20:1019–28. 10.1002/jts.20271 18157881

[25] Chan SS So WK Wong DC Lee AC Tiwari A. Improving older adults' knowledge and practice of preventive measures through a telephone health education during the SARS epidemic in Hong Kong: a pilot study. Int J Nurs Stud. (2007) 44:1120–7. 10.1016/j.ijnurstu.2006.04.019 16857203 PMC7094290

[26] Goodman K Mossad SB Taksler GB Emery J Schramm S Rothberg MB. Impact of video education on influenza vaccination in pregnancy. J Reprod Med. (2015) 60:471–9. 26775454 PMC4827704

[27] Drokow EK Effah CY Agboyibor C Sasu E Amponsem-Boateng C Akpabla GS. The impact of video-based educational interventions on cervical cancer, pap smear and HPV vaccines. Front Public Health. (2021) 9:681319. 10.3389/fpubh.2021.681319 34307280 PMC8294697

[28] Ambe JP Omotara BA Baba MM. Perceptions, beliefs and practices of mothers in sub-urban and rural areas towards measles and measles vaccination in Northern Nigeria. Trop Doct. (2001) 31:89–90. 10.1177/004947550103100211 11321281

[29] Bono SA Faria de Moura Villela E Siau CS Chen WS Pengpid S Hasan MT. Factors affecting COVID-19 vaccine acceptance: an international survey among low-and middle-income countries. Vaccines. (2021) 9:515. 10.3390/vaccines9050515 34067682 PMC8157062

[30] Bester JC. Vaccine refusal and trust: the trouble with coercion and education and suggestions for a cure. J Bioeth Inq. (2015) 12:555–9. 10.1007/s11673-015-9673-1 26626065

[31] Larson HJ Clarke RM Jarrett C Eckersberger E Levine Z Schulz WS. Measuring trust in vaccination: a systematic review. Hum Vaccin Immunother. (2018) 14:1599–609. 10.1080/21645515.2018.1459252 29617183 PMC6067893

[32] Opel DJ Salmon DA Marcuse EK. Building trust to achieve confidence in COVID-19 vaccines. JAMA Netw Open. (2020) 3:e2025672. 10.1001/jamanetworkopen.2020.25672 33079194

[33] Willis DE Andersen JA Bryant-Moore K Selig JP Long CR Felix HC. COVID-19 vaccine hesitancy: race/ethnicity, trust, and fear. Clin Transl Sci. (2021) 1–8. 10.1111/cts.13077 34213073 PMC8444681

[34] Bendau A Plag J Petzold MB Ströhle A. COVID-19 vaccine hesitancy and related fears and anxiety. Int Immunopharmacol. (2021) 97:107724. 10.1016/j.intimp.2021.107724 33951558 PMC8078903

[35] Ishimaru T Okawara M Ando H Hino A Nagata T Tateishi S. Gender differences in the determinants of willingness to get the COVID-19 vaccine among the working-age population in Japan. Hum Vaccin Immunother. (2021) 13:1–7. 10.1080/21645515.2021.1947098 34213406 PMC8827630

[36] Guidry JP Laestadius LI Vraga EK Miller CA Perrin PB Burton CW. Willingness to get the COVID-19 vaccine with and without emergency use authorization. Am J Infect Control. (2021) 49:137–42. 10.1016/j.ajic.2020.11.018 33227323 PMC7677682

[37] Whiteman A Wang A McCain K Gunnels B Toblin R Lee JT. Demographic and social factors associated with COVID-19 vaccination initiation among adults aged≥ 65 years—United States, December 14, 2020–April 10, (2021). Morb Mortal Wkly Rep. (2021) 70:725–30. 10.15585/mmwr.mm7019e4 33983911 PMC8118148

[38] Lazarus JV Ratzan SC Palayew A Gostin LO Larson HJ Rabin K. A global survey of potential acceptance of a COVID-19 vaccine. Nat Med. (2021) 27:225–8. 10.1038/s41591-020-1124-9 33082575 PMC7573523

[39] Byrnes JP Miller DC Schafer WD. Gender differences in risk taking: a meta-analysis. Psychological Bull. (1999) 125:367. 10.1037/0033-2909.125.3.367

[40] Suwantika AA Boersma C Postma MJ. The potential impact of COVID-19 pandemic on the immunization performance in Indonesia. Expert Rev Vaccines. (2020) 19:687–90. 10.1080/14760584.2020.1800461 32758031

